# Supplementary material for: Causal Relationship between Aging and Anorexia Nervosa: A White-Matter-Microstructure-Mediated Mendelian Randomization Analysis
Source: Biomedicines. 2024 Aug 16;12(8):1874. doi: 10.3390/biomedicines12081874 (PMC11351342; doi:10.3390/biomedicines12081874)
Supplement: Supplementary file 1 [file biomedicines-12-01874-s001.zip › Supplementary Figure/Figure S4/Singe SNP plot of MR analysis for TL to ubm-b-1675.pdf]

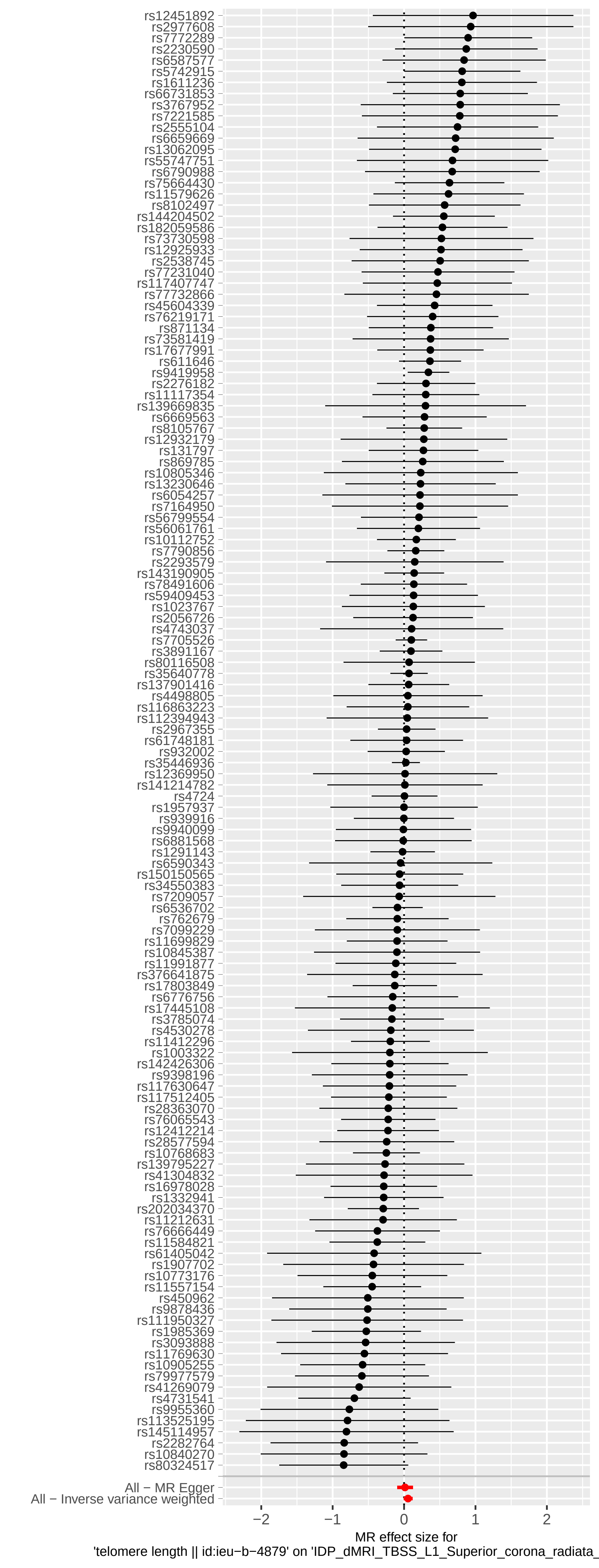
rs12451892
rs2977608
rs7772289
rs2230590
rs6587577
rs5742915
rs1611236
rs66731853
rs3767952
rs7221585
rs2555104
rs6659669
rs13062095
rs55747751
rs6790988
rs75664430
rs11579626
rs8102497
rs144204502
rs182059586
rs73730598
rs12925933
rs2538745
rs77231040
rs117407747
rs77732866
rs45604339
rs76219171
rs871134
rs73581419
rs17677991
rs611646
rs9419958
rs2276182
rs11117354
rs139669835
rs6669563
rs8105767
rs12932179
rs131797
rs869785
rs10805346
rs13230646
rs6054257
rs7164950
rs56799554
rs56061761
rs10112752
rs7790856
rs2293579
rs143190905
rs78491606
rs59409453
rs1023767
rs2056726
rs4743037
rs7705526
rs3891167
rs80116508
rs35640778
rs137901416
rs4498805
rs116863223
rs112394943
rs2967355
rs61748181
rs932002
rs35446936
rs12369950
rs141214782
rs4724
rs1957937
rs939916
rs9940099
rs6881568
rs1291143
rs6590343
rs150150565
rs34550383
rs7209057
rs6536702
rs762679
rs7099229
rs11699829
rs10845387
rs11991877
rs376641875
rs17803849
rs6776756
rs17445108
rs3785074
rs4530278
rs11412296
rs1003322
rs142426306
rs9398196
rs117630647
rs117512405
rs28363070
rs76065543
rs12412214
rs28577594
rs10768683
rs139795227
rs41304832
rs16978028
rs1332941
rs202034370
rs11212631
rs76666449
rs11584821
rs61405042
rs1907702
rs10773176
rs11557154
rs450962
rs9878436
rs111950327
rs1985369
rs3093888
rs11769630
rs10905255
rs79977579
rs41269079
rs4731541
rs9955360
rs113525195
rs145114957
rs2282764
rs10840270
rs80324517
All − MR Egger
All − Inverse variance weighted
−2
−1
0
1
2
MR effect size for
'telomere length || id:ieu−b−4879' on 'IDP_dMRI_TBSS_L1_Superior_corona_radiata_
